# Periostin in lymph node pre-metastatic niches governs lymphatic endothelial cell functions and metastatic colonization

**DOI:** 10.1007/s00018-022-04262-w

**Published:** 2022-05-14

**Authors:** Lionel Gillot, Alizée Lebeau, Louis Baudin, Charles Pottier, Thomas Louis, Tania Durré, Rémi Longuespée, Gabriel Mazzucchelli, Christophe Nizet, Silvia Blacher, Frédéric Kridelka, Agnès Noël

**Affiliations:** 1 Laboratory of Tumor and Development Biology, GIGA-Cancer, Liege University, B23, Avenue Hippocrate 13, Sart-Tilman, 4000 Liege, Belgium; 2 Department of Clinical Pharmacology and Pharmacoepidemiology, Heidelberg University Hospital, Im Neuenheimer Feld 410, 69120 Heidelberg, Germany; 3 GIGA Proteomics Facility, Liège University, Sart-Tilman, 4000 Liège, Belgium; 4 Department of Plastic and Reconstructive Surgery, CHU Liege, Sart-Tilman, 4000 Liege, Belgium; 5 Department of Obstetrics and Gynecology, CHU Liege, Sart-Tilman, 4000 Liege, Belgium; 6 Walloon Excellence in Lifesciences and Biotechnology (WELBIO), Liege University, Liege, Belgium

**Keywords:** Pre-metastatic, Metastatic, Cervical cancer, Lymph node, Periostin

## Abstract

Although lymph node (LN) metastasis is an important prognostic parameter in cervical cancer, the tissue remodeling at a pre-metastatic state is poorly documented in LNs. We here identified periostin (POSTN) as a component of non-metastatic LNs by applying proteomic analyses and computerized image quantifications on LNs of patients with cervical cancer. We provide evidence for remarkable modifications of POSTN and lymphatic vessel distributions and densities in non-metastatic sentinel and metastatic human LNs, when compared to distant non-metastatic LNs. POSTN deposition at a pre-metastatic stage was demonstrated in a pre-clinical murine model (the ear sponge assay). Its expression by fibroblastic LN cells was assessed by in situ hybridization and in vitro cultures. In vitro*,* POSTN promoted lymphatic endothelial cell functions and tumor cell proliferation. Accordingly, the in vivo injection of recombinant POSTN together with VEGF-C boosted the lymphangiogenic response, while the metastatic potential of tumor cells was drastically reduced using a POSTN blocking antibody. This translational study also supports the existence of an unprecedented dialog “in cascade”, between the primary tumor and the first pelvic nodal relay in early cervical cancer, and subsequently from pelvic LN to para-aortic LNs in locally advanced cervical cancers. Collectively, this work highlights the association of POSTN deposition with lymphangiogenesis in LNs, and provides evidence for a key contribution of POSTN in promoting VEGF-C driven lymphangiogenesis and the seeding of metastatic cells.

**Supplementary Information:**

The online version contains supplementary material available at 10.1007/s00018-022-04262-w.

## Introduction

In many cancers, such as cervical, melanoma, and breast cancer, lymph nodes (LN) are the first organs, in which metastases develop and the presence or absence of tumor cells in the draining LN is an important prognostic tool [1–4]. The expression of lymphangiogenic growth factors, high lymphatic vessel density, and a high incidence of lymphatic invasion are typically associated with LN metastasis and reduced survival [5]. Recently, pre-clinical experiments demonstrating the migration of metastatic tumor cells from LN to distant organs have provided the definitive clue that metastatic cells in LN can seed distant organs. This indicates that LN metastases should be treated to prevent distant disease [6, 7].

The concept of pre-metastatic (PM) niche was initially defined by Lyden et al. [8] and refers to a pre-conditioning of the microenvironment of prospective metastatic organs prior to metastatic cell arrival. PM niche formation relies on the secretion of tumor-derived factors such as pro-metastatic growth factors, cytokines, and the release of extracellular vesicles [9, 10]. This organ pre-conditioning supports the subsequent survival and proliferation of disseminating tumor cells. The recruitment of specific cell types [11] and the remodeling of the extracellular matrix (ECM) are therefore seen as crucial events in PM elaboration. PM niches have been well described for lung, liver and bone [12–14], but far less for LNs [15]. Lymphatic vasculature remodeling (lymphangiogenesis) is a key step in the formation of a LN PM niche and is primarily driven by vascular endothelial growth factors (VEGF-A and VEGF-C) [16, 17]. The existence of a pre-metastatic dialog between the primary tumor and the first nodal relay is supported by translational studies [1, 13, 18]. Both lymphatic and immune responses contribute to the elaboration of a specific microenvironment in human non-metastatic sentinel LNs [2]. Our previous work conducted in the context of early cervical cancer, demonstrated that lymphangiogenesis occurs within the sentinel LNs and is associated with a specific humoral immune response [2].

ECM degradation and deposition, which are often associated with tissue remodeling are poorly documented in LN PM [15]. One ECM component, periostin (POSTN) plays a key role by interacting with other components such as fibronectin, tenascin-c (TNC) and collagen types I, IV and V [19]. POSTN over-expression has been observed in several cancer types including breast, lung, colorectal, ovary and prostate cancers [20]. POSTN has also been successfully identified as a component of lung and liver PM niches [21, 22]. Notably, organ specificities have been noticed in matrix remodeling associated with PM niche [23]. POSTN has been detected in metastatic LNs of squamous cervical cancer [24]. However, its role in the elaboration of a PM in LNs and its putative functions in lymphangiogenesis and LN colonization by metastatic tumor cells still need to be elucidated. Here, by combining translational and pre-clinical settings, we identified POSTN as a crucial fibroblasts-derived matricellular protein implicated in the LN PM niche development that regulates lymphatic endothelial cell (LEC) functions and promotes metastatic cell implantation in LNs. Analysis of sentinel, non-metastatic and metastatic LNs issued from early and locally advanced cervical cancers, provides the first evidence for a cross-talk “in cascade” between the primary tumor and the first pelvic nodal relay in early cervical cancer, and subsequently from pelvic LN to para-aortic LNs in locally advanced cervical cancers.

## Materials and methods

### Human tissue samples

Regarding early cervical cancer (FIGO stage 1B1), pelvic LNs were obtained from 38 patients as previously described [2]. The patients were divided into two groups according to the presence (“metastatic”) or absence (“non-metastatic”) of metastatic cells in sentinel LNs. In the group of non-metastatic patients (26/38), one sentinel pelvic lymph node (SLN-) was selected and one distant pelvic lymph node (DLN-) was chosen from the 13 other patients. The second group consisted of patients diagnosed with at least one metastatic pelvic LN (MLN+).

Regarding locally advanced cervical cancer (FIGO stage 1B1 to 2B), para-aortic LNs were surgically collected from 45 patients (3 LNs per patients). Patients were subjected to positron emission tomography (PET scan) to determine the status (positive or negative) of pelvic (LNP) and para-aortic (LNAo) LNs. Three groups of patients were defined. In the first group, patients had no pelvic or para-aortic LN metastasis (LNP−/LNAo−) (19/45). The second group consisted of patients diagnosed with at least one metastatic pelvic LN, but no para-aortic metastasis (LNP+/LNAo−) (13/45). In the third group, patients were diagnosed with at least one metastatic pelvic LN and one metastatic para-aortic LN (LNP+/LNAo+) (12/45). In this group, one metastatic para-aortic LN (LNP+/LNAo+M+) and 3 non-metastatic para-aortic LN were selected (LNP+/LNAo+ M−). All tissues were reviewed by pathologists.

### Immunohistochemical stainings of human samples

Formalin-fixed tissue sections were analyzed by immunofluorescence using an EnVision kit (Dako K4003). After deparaffinization, the sections were microwaved in citrate buffer (Dako S2369) for 11 min, at 126 °C. Endogenous peroxidases were blocked with 3% H_2_O_2_ (8070-4, Roth). Sections were blocked in animal-free blocking solution (15019 L, Cell Signaling) and incubated with the primary antibody. The EnVision-HRP secondary antibody was incubated at room temperature for 30 min. The tyramide signal amplification (TSA) (NEL741001KT, Akoya Biosciences) working buffer, containing fluorescein isothiocyanate (FITC) or Cyanin 3, was used for signal amplification. After removing the primary and secondary antibodies by microwaving the slides, the same procedure was repeated with the next primary antibody and TSA working buffer. The primary antibodies included a rabbit anti-POSTN (1/100, HPA012306, Sigma) and mouse anti-D2-40 (1/200, M3619, Dako) to detect lymphatic vessels. All the slides were mounted with fluoromount containing DAPI (SouthernBiotech 0100-20). Sections were digitalized using NanoZoomer 2.0-HT system (0.23 µm/pixel (× 20) scanning resolution). A minimum of 12 images per experimental condition were chosen and used for computerized quantification.

### Proteomic analyses on human samples

A microproteomic workflow was followed for biomarker discovery, as previously described [25, 26]. Serial sections of 4–5 μm from formalin-fixed and paraffin-embedded (FFPE) tissue blocks from 6 control and 5 sentinel LNs were cut. For each specimen, a first section was used for hematoxylin/eosin (HE) staining, scanning, and histological evaluation and a serial section was used for laser micro-dissection [25, 26]. Cytomine software [27, 28] was used to calculate the surfaces of the sub-capsular sinus containing lymphatic vessels, and to determine the number of immune cells. In order to avoid artefacts in proteomic results due to the presence of different proportions of immune cells between samples, selected tissue areas containing the closest ratio (tissue area/number of immune cells) between samples were targeted. Considering all samples, average tissue surfaces of approximately 811,000 µm^2^, containing an average number of 3700 immune cells, resulting in an average ratio (tissue area/number of immune cells) of 222 (SD = 55). The identified regions of interest were laser micro-dissected and processed for microproteomics, as described before [25, 26].^.^Data processing was performed using MaxQuant version 1.5.2.8 for label-free quantification (LFQ) [29]. Statistical analyses of data were carried out using Perseus software version 1.5.0.15 [30], as previously described [26, 31]. For biomarker discovery, *t* test analyses were performed. Only entries for which quantification was possible in at least four samples in one group (control or sentinel) were kept. The following settings were used for *t* test: s0 = 1, permutation based false discovery rate (FDR) with FDR of 0.05.

### In situ hybridization

The mRNA in situ hybridization of POSTN was measured on human LN tissue sections using the RNAscope assay according to manufacturer’s instructions (Advanced Cell Diagnostics, Bioké, Leiden, The Netherlands). In brief, tissue Sects. (10 μm) were dehydrated and hybridized with Hu-POSTN-C2 (#409181-C2, Bioké, Leiden, The Netherlands) probes or with a RNAscope 3-plex negative control probe (#320871, Bioké, Leiden, The Netherlands). Hybridization signal was amplified with RNAscope Multiplex Fluorescent reagent kit V2 (#323135, Bioké, Leiden, The Netherlands). After hybridization, the sections were incubated for 1 h with anti-αSMA-FITC antibody (F3777, Sigma). Images were generated with a confocal Zeiss HR microscope (Zeiss, Germany) and a 63 × objective lens.

### Mice

C57Bl6 mice or Swiss Nude mice (both 6 to 8 weeks-old) were used throughout this study. The animals were maintained under a 12-h light–dark cycle with free access to food and water.

### Ear sponge assay

Gelatin sponges were incubated with either tumor cells (2 × 10^5^ B16F10 cells or 6 × 10^6^ CaSki cells/sponge) or control medium (serum-free DMEM without tumor cells) for 30 min in serum free-DMEM, embedded with collagen and implanted into mouse ears as previously described [32, 33]. Bioluminescence was detected in animals bearing ear sponges soaked with luciferase expressing cells using the in vivo Imaging System IVIS 200 (Xenogen Corp.; Alameda, CA, USA). At the end of the assay, cervical LNs were removed, embedded in tissue OCT (Tissue-Tek) and frozen at − 80 °C. In some assays, anti-POSTN antibody (Adipogen AG-20B-6000PF) or control IgM (0.25 µg/µl) (Adipogen ANC-290-810) was injected directly into the sponge, twice a week, for a period of 4 weeks with a Hamilton syringe. At the end of the experiment, the sponges and LNs were harvested, incubated in 4% formol (11699408, VWR) for 4 h, dehydrated in ethanol and fixed in paraffin (X881.2, Leica).

### Intra-nodal injection

Blue Evans 2% (5 µl) was injected into a fat pad to identify the inguinal LN. After 30 min, B16F10 cells (3 × 10^5^) were injected in the inguinal LN with anti-POSTN antibody or IgM Ctrl using a microsyringe (Hamilton). In some assays, 1 µl of recombinant POSTN (100 µg/ml) and/or VEGF-C (200 µg/ml) were also injected in the inguinal LN. At day 3 post-injection, the LNs were removed and embedded in paraffin.

### Immunohistochemical stainings of murine samples

Cryosections (5 µm) were fixed with 4% formol (11699408, VWR) for 10 min and permeabilized for 5 min with 1% Triton X-100 (108603, Millipore) at room temperature. Sections were blocked for 20 min in Animal-Free blocking solution (15019 L, Cell signaling) and incubated for 1 h at room temperature with antibodies raised against POSTN (1/500; Adipogen AG-20B-0033B) and LYVE1 (1/200; AF2125, R&D Systems). After washing in PBS, anti-streptavidin Alexa Fluor 555 (1/500; S21381, Invitrogen) and Donkey anti-goat Alexa Fluor 488 (1/200; A11055, Invitrogen) were added for 30 min at room temperature. For HEV detection, sections were blocked with an animal-free blocking solution and incubated overnight at 4 °C with a rat anti-MECA79-Alexa Fluor 488 antibody (53-6036-82, Invitrogen).

Paraffin sections were analyzed using an immunofluorescence EnVision kit (Dako K4003). After deparaffinization, the sections were microwaved in citrate buffer (Dako S2369) for 11 min at 126 °C. Endogenous peroxidases were blocked with 3% H_2_O_2_ (8070-4, Roth). Sections were blocked in an animal-free blocking solution and incubated with the primary antibody. The EnVision-HRP secondary antibody was incubated at room temperature for 30 min and a TSA working buffer containing FITC or Cy3 was used for signal amplification. After removing the primary and secondary antibodies by microwaving the slides, the same procedure was repeated with the next primary antibody and TSA working buffer. Primary antibodies included a mouse anti-POSTN (1/500), a rat anti-HEV/HRP (1/50, Sc19602, Santa Cruz) and LYVE1 (1/200). All slides were mounted with fluoromount with DAPI. For tumor cell detection, a Fontana staining was performed. In short, sections were treated with ammoniacal silver solution for a period of 12 h. After washing in distilled water, gold chloride was added for a period of 4 min. Subsequently, sections were fixed in sodium hyposulfite, counterstain with nuclear red stain dehydrated and then mounted. Sections were digitalized using a NanoZoomer 2.0-HT system [0.23 µm/pixel (× 20) scanning resolution]. A minimum of 30 images per experimental condition were used for computerized quantification.

For 3D images, LNs were fixed in formol 4% for 2 h and permeabilized for a period of 1 day with 2% Triton X-100 in PBS solution containing 0.05% sodium azide. LNs were blocked for 2 days with animal free blocking solution and then incubated for 2 days with primary antibodies (POSTN and LYVE1). After washing in PBS, anti-streptavidin Alexa Fluor 555 and Donkey anti-goat Alexa Fluor 488 were added for 1 day at room temperature. Then, LNs were incubated with RapiClear 1.52 (RC152001, SunjinLab) a clearing solution for 2 days. LNs were digitalized on Zeiss LSM880 confocal microscope at 10 × magnification.

### Cell culture and reagents

Primary human LECs (HMVEC-dLy from Lonza, Belgium) were cultured in EGM2-MV medium until confluence. Primary human lymphatic fibroblasts (HLF from ScienCell, referred to as FRC in the text) were cultured in complete Fibroblast Growth Medium, until confluence was achieved, according to the manufacturer’s instructions. Murine B16F10Luc melanoma cells and human cervical CaSki cells were purchased from Caliper Lifesciences and American Type Culture Collection (ATCC), respectively. Tumor cells were maintained in Dulbecco’s modified Eagle’s medium (DMEM, 10938-025, Thermofisher, MA) supplemented with 10% fetal bovine serum (10270-106, Thermofisher), 1% glutamine (25030-123, Thermofisher) and 1% penicillin–streptomycin (15140-122, Thermofisher).

Cells were stimulated with recombinant human VEGF-C (400 ng/ml) (Abcam 155739), medium conditioned by cells (see below) or serum-free medium as a control. For the preparation of medium conditioned (CM) by cells (tumor cells), subconfluent tumor cells were incubated in serum-free DMEM for 48 h. CM was harvested, centrifuged at 1000×*g* for 10 min and concentrated 10 × with Amicon Ultra Centrifugal Filters 3 K (UFC900324, Millipore). CM aliquots were stored at − 20 °C until use.

For phalloidin staining, LECs or B16F10 were fixed in 1% paraformaldehyde in PBS at room temperature for 10 min. Cells were then incubated with phalloidin-Atto 550 for 1 h at room temperature. Fluoromount with DAPI was used to mount the coverslips. Image acquisition was performed on an epifluorescence microscope (Apotome Zeiss 40 × objective lens).

### Immunohistochemistry on cellular-derived matrix

24 well plate coverslips were coated with 0.2% gelatin for 1 h at 37 °C. After washing in PBS, 1% of glutaraldehyde was added for 30 min. Then, ethanolamine (1 M) was used for 30 min at room temperature to block the remaining glutaraldehyde. FRC were seeded (1 × 10^5^) and cultured for 6 days. At day 1, 3 and 6, the medium was removed and the cells stimulated with CM from tumor cells or VEGF-C 400 ng/ml. At day 7, the cells were fixed with 4% paraformaldehyde and blocked for a period of 1 h at room temperature with BSA 1%. Primary antibody against POSTN (1/200; Adipogen) was added and the cells were incubated overnight. After washing in PBS, anti-streptavidin Alexa Fluor 555 (1/500; S21381, Invitrogen) was added and they were left for 30 min at room temperature. Coverslips were then mounted with fluoromount with DAPI. Images were obtained using an epifluorescence microscope (Apotome Zeiss 40 × objective lens).

### Proliferation assay

96-well-plates were coated with POSTN (10 ng/well) (R&D systems) or water overnight at room temperature. Tumor cells (1 × 10^4^) or LEC (2 × 10^4^) were seeded in a 96-well plate for 24 h or 48 h. Cells were cultured in serum-free medium and cellular growth was measured over a 24 h (for LEC) or 48 h (for tumor cells) period using the IncuCyte S3 Live-Cell Analysis System (Essen Biosciences) at 10 × magnification. Data were analyzed using the IncuCyte analysis software (IncuCyte S3 2019A, Essen Biosciences).

### Adhesion assay

After POSTN coating (described above), LECs or B16F10 were stained with 1 µm calcein (Invitrogen) in a serum-free medium, for 15 min at 37 °C. After washing with PBS and incubating with medium for 1 h to remove excess dye, cells were detached with mild trypsinization. LECs or B16F10 (2 × 10^4^/well) were allowed to adhere to the matrix for 1 h at 37 °C, then washed twice with PBS. Adherent cells were detected at 485 nm excitation and 538 nm emission wavelength using a fluorescence plate reader (SpectraMax, Molecular Devices).

### ELISA

POSTN concentration, in the indicated culture medium (24 h after stimulation), was determined by ELISA according to the manufacturer’s recommendation (Human DuoSet Periostin, DY3548B, R&D systems).

### Directional migration assay

LECs or B16F10 (2 × 10^4^) suspended in EGM-2 MV or DMEM complete medium were first allowed to adhere for 4 h in a μ-slide chemotaxis 2D assay (80326, Ibidi). After washing, cells were then maintained in a serum-free medium, and a chemoattractant was added to the upper chamber (100 ng of Human Recombinant POSTN or complete medium). Cell migration was observed for 20 h with a Nikon A1R confocal microscope and a 10 × objective lens. Analyzes of velocity (µm/min) and accumulated distance (µm) were performed using Ibidi software (Ibidi) as previously described [34, 35].

### qRT-PCR

After RNA extraction (High Pure RNA Isolation Kit, Roche) and reverse transcription (First Strand cDNA Synthesis kit, Roche), real-time PCR was performed with the LightCycler480 Probes Master kit and the Universal Probe Library system (Roche). The following primers were used: 5′-TTGCAGATCATCAAGAACACGTAGA-3′ (forward primer) and 5′-CAGTAGAGATCAGTTGTCTCTGGTTGC-3′ for E6–E7.

### Statistical analysis

Statistical analysis was performed with GraphPad Prism 6.0 software using the Mann–Whitney test, two-tailed (not assuming Gaussian distributions), Chi-square test- or two-way ANOVA, as indicated in the figure legends. Data are shown as mean ± SD and differences were considered statistically significant when *p* < 0.05, as indicated by asterisks with *p* < 0.05 (*), *p* < 0.01 (**), *p* < 0.001 (***) and *p* < 0.0001 (****).

### Study approval

Animal experiments were performed in compliance with the Animal Ethical rules of the University of Liège (Liège, Belgium) after approval from the local Animal Ethical Committee. Human LN samples were stored in the biobanks of the University of Liège (CHU, Liège, Belgium) after study approval by local ethic committees.

## Results

### POSTN is upregulated in sentinel LNs from patients with early cervical cancer

**Fig. 1:**
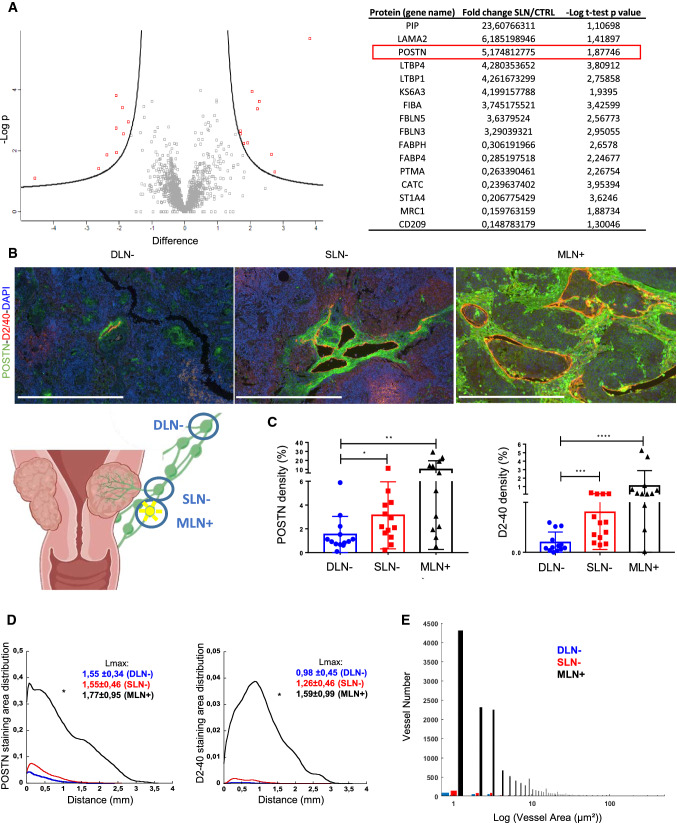
Periostin is upregulated in sentinel LNs from patients with early cervical cancer.** a** The marginal sinus of LN from patients with a cervical carcinoma has been laser-micro dissected (*n* ≥ 5). Protein extracts were subjected to proteomic analysis using mass spectrometry. Volcano plot of the proteomic analysis. Volcano plot based on the mean of the protein fold change associated with its *P* value (− Log*P*). Each red dot corresponds to a statistically significant protein between the sentinel and the control LN. Proteins modulated between the sentinel and the non-sentinel LN are indicated in the table. **b** Double immunostaining of POSTN (green), Podoplanin (D2-40 in red) and nuclei (DAPI, blue) on human LNs: distant negative LNs (DLN−, *n* = 13), negative sentinel LN (SLN−, *n* = 13) and metastatic LNs (MLN+, *n* = 12). Scale bars represent 500 µm. **c** Computer-assisted quantification of POSTN and Podoplanin densities (in percentage) in sentinel and metastatic LNs. Graphs are presented as scatter plots of individual data points. Results are expressed as mean ± SD (Wilcoxon–Mann–Whitney test: p* < 0.05; p** < 0.01; p*** < 0.001; p**** < 0.0001). **d** Spatial lymphatic vessel and POSTN distribution from tissue edge to tissue center measured on whole tissues of DLN−, SLN− and MLN+ (statistical analyses: Kolmogorov Smirnov test, *p < 0.05). Maximum distance of migration from the tissue border (Lmax) is indicated and expressed as mean ± SD. **e** Lymphatic vessel size distribution

A mass spectrometry-based proteomic analysis was performed on the micro-dissected sub-capsular sinus of sentinel (*n* = 5) and non-sentinel (*n* = 6) LNs from patients diagnosed with early cervical cancer (FIGO stage IB1). A total of 1784 proteins were detected, of which only 16 proteins were differentially detected in the two sample groups: 9 with higher levels (> threefold-change) and 7 with lower levels in SLNs (Fig. 1a). They mainly included ECM components (laminin, POSTN) and associated proteins (fibulin or FBLNs and latent-transforming growth factor binding proteins or LTBPs). POSTN was in the top 3 of the upregulated proteins with a fold change of 5.17 (Fig. 1a). To validate this modulation, the POSTN was then analyzed using immunohistochemical (IHC) staining on LNs from patients with early cervical cancer (*n* = 38). Metastatic cell-free sentinel LNs (SLN−), metastatic cell-free distant pelvic LNs (DLN−) and metastatic cell positive LNs (MLN+) were subjected to double immunostaining of POSTN and podoplanin (D2/40 antibody) to detect lymphatic vessels as proof of lympangiogenesis (Fig. 1b). Sparse POSTN staining was observed in DLN−, while denser POSTN deposits were seen in SLN− and MLN+. Complete slide computer-assisted quantifications [36] revealed increased POSTN density in SLN− and MLN+ in comparison with DLN− (Fig. 1c). In line with a previous report [2], lymphatic vessel density was also higher in SLN− and MLN+ (Fig. 1c). Interestingly, lymphatic vessels were localized in those POSTN positive areas (Fig. 1b). The spatial distributions of lymphatic vessels and POSTN were then compared. They were similar in both DLN− and SLN− with a localization of both POSTN and lymphatic vessels in the LN border (at a distance of less than 1.5 mm from the tissue edge) (Fig. 1d). In sharp contrast, in MLN+, an enlarged peak of the distribution curve was observed which reflected the infiltration of POSTN and lymphatic vessels deeper in the LN. In line with this global analysis, the maximum distance from the LN border was similar in DLN− (POSTN: 1.55 ± 0.34 mm; D2-40: 0.98 ± 0.45 mm) and SLN− (POSTN: 1.55 ± 0.46 mm; D2-40: 1.26 ± 0.46 mm), but tended to be higher in MLN+ (POSTN: 1.77 ± 0.95 mm; D2-40: 1.59 ± 0.99 mm) (Fig. 1d). The size of the lymphatic vessels was also measured with MLN+ (Fig. 1e) showing an increase in size. Collectively, these data denoted early modifications of POSTN deposition and lymphatic vessel expansion in the capsular and sub-capsular areas of SLN− before any detection of metastatic cells. Metastatic LNs are characterized by a densification of POSTN and lymphatic networks with a deep infiltration into the central area of the organ.

### POSTN is upregulated in (pre)-metastatic LN and is associated with lymphatic vessels

**Fig. 2:**
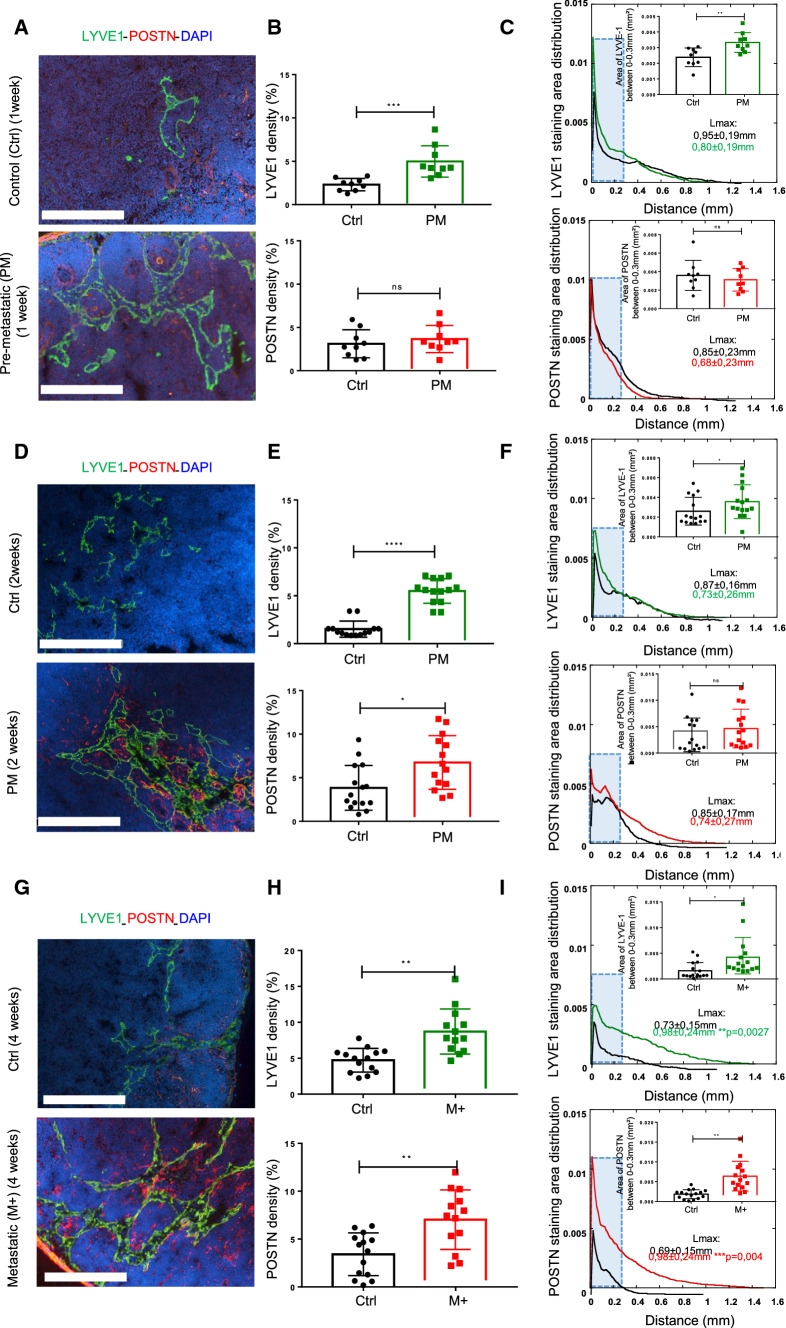
POSTN is upregulated in (pre)-metastatic LN*.* Morphometric analysis of POSTN and LYVE1+ lymphatic vessels in experimental (pre)-metastatic LN (in the ear sponge assay using B16F10 cells). CTRLs correspond to mice implanted with a sponge without tumor cells. **a**–**d**–**g** Immunostaining of POSTN (red) and LYVE1 (green) in pre-metastatic (PM) (at 1 week in A, at 2 weeks in **d**) and in metastatic (M+) LNs (G). Bars = 250 µm. **b**–**e**–**h** Scatter graphs use scatter plots to represent POSTN and LYVE1 densities (in percentage) assessed by a computer assisted method (*n* ≥ 9). Results are expressed as mean ± SD, and statistical analyses were performed using a Wilcoxon–Mann–Whitney test (*p < 0.05; **p < 0.01; ***p < 0.001; ****p < 0.0001). **c**–**f**–**i** Spatial distribution analysis from tissue edge to tissue center. The blue rectangle indicates the area between 0–0.30 mm from the LN border where the cumulate normalized areas of LYVE1 and POSTN were measured and represented in the top right. Maximum distance of migration from the tissue border (Lmax) is indicated. Results are expressed as mean ± SD (Wilcoxon–Mann–Whitney test: *p < 0.05; **p < 0.01). **d**–**i** All the results represent the set of two independent experiments

Temporal investigation of POSTN and lymphatic vasculature remodeling in LNs, was carried out using the pre-clinical murine ear sponge assay that reproduced the different steps of LN metastatic dissemination [32, 33, 37]. Due to the lack of authentic murine cervical cancer cell line, melanoma B16F10Luc cells were used as a model of LN metastatic cells [32]. Gelatin sponges soaked with tumor cells were transplanted into the ears of mice leading to the formation of a local tumor but with no detection of metastatic cells during the two first weeks (pre-metastatic stage: PM) (Fig. 2a, d). At 4 weeks, however, metastases were observed in the LNs (metastatic stage: M+) (Fig. 2G). The presence or absence of metastases at 2 and 4 weeks was assessed by LUC+ signal and by immunohistochemistry as previously described [32]. Gelatin sponges devoid of tumor cells were used as controls (CTRL). To stain POSTN and LYVE1+ lymphatic vessels, LNs were subjected to double IHC and at 1 week, global POSTN density was seen to be similar in both PM and CTRL LNs (Fig. 2a, b). In sharp contrast, the global POSTN density was increased roughly 2 to threefold at 2 and 4 weeks post-implantation (Fig. 2e, h). The global lymphatic vessel density was increased at 1, 2 and 4 weeks post implantation (Fig. 2b, e, h), suggesting that lymphangiogenesis had already been initiated before the deposition of POSTN. In line with our clinical data and by comparing the spatial distribution curves of POSTN and lymphatic vessels, no change was detected at an early PM stage (1 week) (Fig. 2c). However, a densification and a deeper infiltration of the POSTN network were found at a latter PM stage (week 2) inside the LN and at the M stage (week 4) (Fig. 2f, i). Although there was no difference in the global curve at PM stages in the lymphatic vessels, (Fig. 2c, f), they were seen to migrate deeper toward the center of metastatic LNs (Fig. 2I). In line with clinical data, this spatial distribution analyzes revealed a higher peak of POSTN and LYVE1 at the LN periphery (Fig. 2c, f, i). A more in-depth examination of POSTN and LYVE1 densities in the LN border, i.e. between 0 and a maximum of 0.30 mm from the tissue edge, once again revealed an increased density of LYVE1 at all stages, and of POSTN at 4 weeks (Fig. 2c, f, i).

**Fig. 3:**
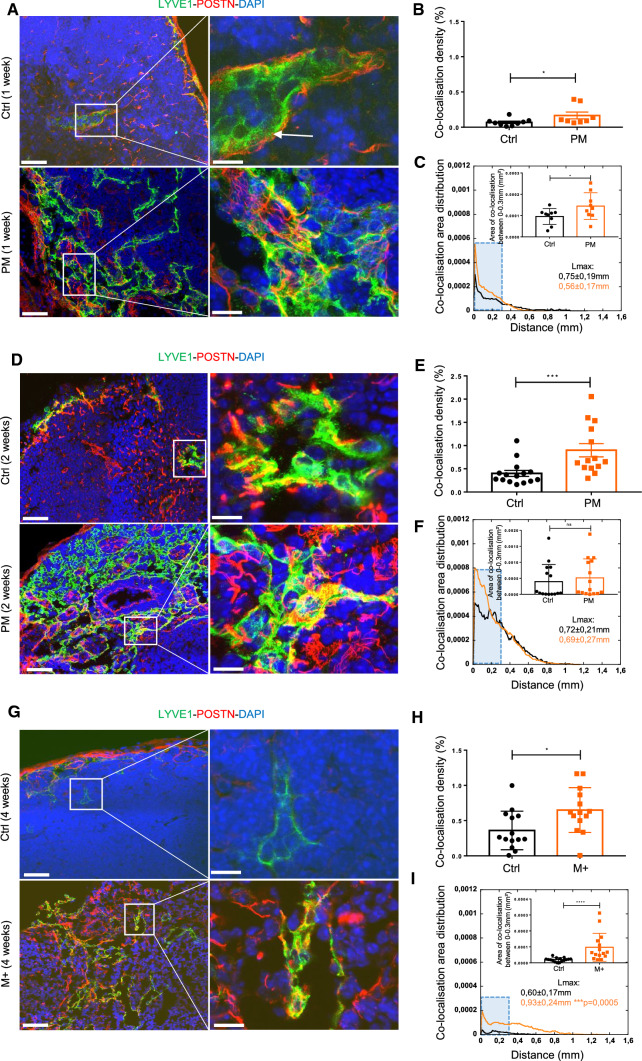
POSTN is associated with lymphatic vessels. Morphometric analysis of experimental (pre)-metastatic LNs as described in Fig. 2. **a**–**d**–**g** Colocalization analysis of POSTN (red) and LYVE1 (green) at PM (at 1 week in A and at 2 weeks in **d**) and M (at 4 weeks in **g**) stages. Bars = 50 µm and 10 µm in the right (higher magnification of the insert) images. **b**–**e**–**h** Scatter graphs use scatter plots to represent POSTN-LYVE1 colocalization densities (in percentage) (*n* ≥ 7). Results are expressed as mean ± SD (Wilcoxon–Mann–Whitney test: *p < 0.05; **p < 0.01; ***p < 0.001). **c**–**f**–**i** Spatial distribution analysis from tissue edge to tissue center. The blue rectangle delineates the area between 0–0.30 mm from the LN border where the cumulate normalized area of LYVE1 and POSTN were measured and represented in the top right. Maximum distance of migration from the tissue border (Lmax) is indicated. Results are expressed as mean ± SD (Wilcoxon–Mann–Whitney test: *p < 0.05; **p < 0.01; ***p < 0.001; ****p < 0.0001)

Interestingly, a colocalization between LYVE1 and POSTN was observed during the (pre)-metastatic process (Fig. 3a, d, g). This colocalization density was weak at 1 week, but increased over time (at 2 weeks) and was higher under metastatic conditions (Fig. 3b, e, h). The distribution of colocalization was also higher in (pre) and metastatic conditions (Fig. 3c, f, i). The analyzes of colocalization between 0 and 0.30 mm from the LN border revealed differences at 1 and 4 weeks (Fig. 3c, i). A deeper infiltration of POSTN (0.98 ± 0.24 mm in M + vs 0.69 ± 0.15 mm in CTRL; **p = 0.004) (Fig. 2i), LYVE1 (0.98 ± 0.24 mm in M + vs0.73 ± 0.15 mm in CTRL; **p = 0.0027) (Fig. 2i) and the colocalization (0.93 ± 0.24 mm in M + vs 0.60 ± 0.17 mm in CTRL; ***p = 0.0005) (Fig. 3i) was found at the metastatic stage, as measured by the maximum migration distance determination. Accordingly, our data showed an increase of POSTN and LYVE1 lymphatic vessels in (pre)-metastatic LNs.

This observation was further illustrated by 3D images performed on the complete LN (Supplementary Fig. 1).

All these results were confirmed using an additional cancer cell type: human cervical cancer CaSki cells (Supplementary Fig. 2). To detect the presence or absence of CaSki cells in mouse LN, qRT-PCR on E6-E7 HPV proteins was performed with the primary tumor being used as the positive control. All LNs were found to be negative for tumor cells (Ct ≥ 40 cycles) and, therefore, considered as a PM stage.

### POSTN is not associated with HEVs in (pre)-metastatic LNs

To determine whether the relationship between POSTN and lymphatics could be restricted to this vascular system, MECA79 staining was compared to identify high endothelial veinules (HEVs), which can act as a portal allowing metastatic cells to enter blood vessels and exit LNs (Supplementary Fig. 3A, D, G). HEV density was increased in LNs of mice bearing tumors, independently of the stages (1, 2 and 4 weeks) (Supplementary Fig. 3B, E, H). In contrast to what was found for lymphatic vessels, HEV and POSTN stainings were not connected in any way. The spatial distribution curve also aided higher HEV density in PM and M conditions (Supplementary Fig. 3C, F and 4). At 2 weeks post implantation, HEV were found deeper in the LN center (± 0.70 mm versus ± 1 mm; *p < 0.05) and a higher peak of HEV was observed on the periphery of the LN. Analysis of HEV from the LN border (between 0 and 0.40 mm) showed a difference at all stages (Supplementary Fig. 3C, F, I).

The presence of HEVs was detected inside the tumor area and in uncolonized LN areas (Supplementary Fig. 4).

### POSTN is produced by fibroblasts in LNs

To determine the cellular source of POSTN, in situ hybridization was performed on human LN samples (early cervical cancer). POSTN mRNA was expressed by α-SMA positive cells (Supplementary Fig. 5A). Following this, POSTN production by ELISA was determined in in vitro cultures of FRCs and LECs either stimulated with VEGF-C (400 ng/ml) or with medium conditioned by B16F10 or CaSki cells (Supplementary Fig. 5B). Notably, POSTN was not detected in the tumor cell conditioned medium. Under basal conditions, FRCs secreted 24-fold more POSTN in comparison with LECs (30,165 pg/ml in FRC vs 1256 pg/ml in LEC) (Supplementary Fig. 5B). POSTN secretion was elevated in FRC stimulated by tumor cell conditioned medium (B16F10 and CaSki) and VEGF-C, when compared to the control FRC. The low amounts of POSTN secreted by LECs were unaffected by both VEGF-C and medium conditioned by tumor cells. Immunostaining of confluent FRC was performed to determine how efficiently cells deposited POSTN into a matrix. After 7 days in culture, a uniform matrix was perceived and an increase in POSTN density observed upon VEGF-C and tumor cell conditioned medium stimulation (Supplementary Fig. 5C).

### POSTN exerts different effects on LECs and tumor cells

The detection of colocalization between POSTN and lymphatic vessels in clinical and pre-clinical LN samples led to an assumption that POSTN could contribute to the expansion of the lymphatic network deeper inside the LN. Furthermore, POSTN could modulate the implantation of metastatic cells by promoting their proliferation and/or migration. The ability of POSTN to support the adhesion and proliferation of LEC and tumor cells was investigated by using recombinant POSTN. Cells (LEC or tumor cells) dyed with calcein, were seeded on POSTN and left for 1 h after which the percentage of adherent cells was estimated by fluorescence measurement. An increase in LEC adhesion was observed in the POSTN-coated well, although POSTN had no effect on B16F10 cell adhesion (Supplementary Fig. 6A). In contrast, POSTN promoted both LEC and B16F10 proliferation after 24 and 48 h of culture on the POSTN-coated well, respectively (Supplementary Fig. 6B). Cells were stained with phalloidin antibody to analyze the impact of POSTN on LEC and B16F10 morphology (Supplementary Fig. 6C). Computerized-analysis was used to measure the area (µm^2^) and circularity of LEC. The circularity of an object is defined as the ratio between its area and its perimeter and a circularity value of 1 indicates a perfect circle, while values close to 0, reflect an increasingly elongated form. The area of LEC was higher (Supplementary Fig. 6C) and less circular (Supplementary Fig. 6C) on the POSTN-coated dishes. However, no impact of POSTN on tumor-cell morphology was noticed. The impact of POSTN on LEC and B16F10 migration was also studied by using the microslide-chemotaxis model from IBIDI. In brief, cells were seeded in a channel and a POSTN gradient (100 ng/ml) or serum gradient was created. POSTN promoted the directional migration of LECs (Supplementary Fig. 6D), but not that of B16F10 cells. The velocity and accumulated distance of LECs between serum and POSTN conditions (Supplementary Fig. 6D) were found to be the same.

### POSTN blocking antibody reduces LN invasion in vivo

**Fig. 4:**
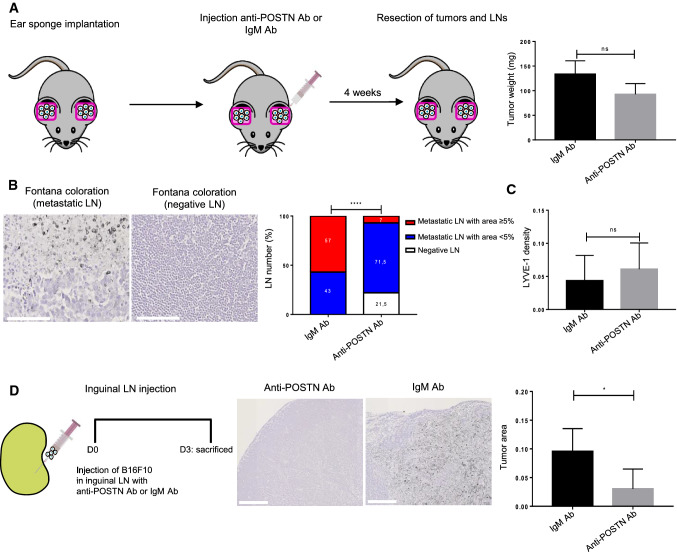
POSTN blocking antibody reduces LN invasion in vivo.** a** Schematic representation of the ear sponge assay. POSTN blocking antibody (Ab) or IgM Ab (ctrl) was injected into the sponge soaked with B16F10, twice a week, for a period of 4 weeks. The histogram represents the tumor weight (mg) at the end of the assay (*n* ≥ 15). **b** Fontana staining to detect tumor cells in a positive (metastatic, left) and negative (right) LNs. Bars = 100 µm. Histogram represents the metastatic index: percentage of metastasis-free LN (negative LNs in white), positive LNs with a metastases area < 5% (low metastatic index in blue) and positive LNs with metastases area ≥ 5% (high metastatic index in red) in both groups (anti-POSTN Ab and IgM Ab). Statistical analyses were performed using Chi-square (****p < 0.0001) **c** Morphometric analysis of LYVE1 lymphatic vessels in LNs treated with anti-POSTN or IgM Ab. The scatter graph uses dots to represent LYVE1 density assessed by a computer assisted method. Results are expressed as mean ± SD (Wilcoxon–Mann–Whitney test). **d** Schematic representation of the intra-nodal injection of B16F10 cells (with anti-POSTN or IgM) using a Hamilton syringe. Fontana staining to detect tumor cells in LNs treated with anti-POSTN or IgM Ab. Bars = 250 µm. Histogram represents the tumor area (surface occupied by tumor cells divided by total surface of LN). Results are expressed as mean ± SD (*n* ≥ 6) (Wilcoxon–Mann–Whitney test (*p < 0.05)

To determine the impact of POSTN on LN metastases, POSTN-blocking antibody or IgM ctrl was injected into the ears of tumor bearing mice, twice a week for a period of 4 weeks (0.25 µg/µl). No impact on the primary tumor weight was detected (Fig. 4a). In mice treated with an anti-POSTN antibody, the percentage of non-metastatic LN assessed by IHC (Fontana staining) was 21.5%, while 100% of IgM-treated mice developed metastasis (Fig. 4b). The LNs were then classified according to the metastatic index (LN metastatic area/total LN area): metastasis-free (negative LNs), low metastatic index (less than 5% of the global surface) and high metastatic index (more than 5% of total LN area). The metastatic index in the anti-POSTN treated group was smaller, with 21.5% of metastasis-free LNs and only 7% of LNs with a high metastatic index. In the control group, however, 57% of LNs displayed a high metastatic index. No differences in LYVE1 staining were observed in either experimental group (Fig. 4c). To confirm the impact of POSTN blocking antibody on LN colonization, a second mouse model was used. This time, B16F10 cells were directly injected with a micro-syringe into the inguinal LN in the presence of a POSTN blocking antibody or IgM ctrl (Fig. 4d). Three days post-injection, a significant reduction of tumor area was detected in LNs of mice injected with tumor cells and anti-POSTN antibody (Fig. 4d).

### POSTN boosts in vivo the lymphangiogenic response initiated by VEGF-C

**Fig. 5:**
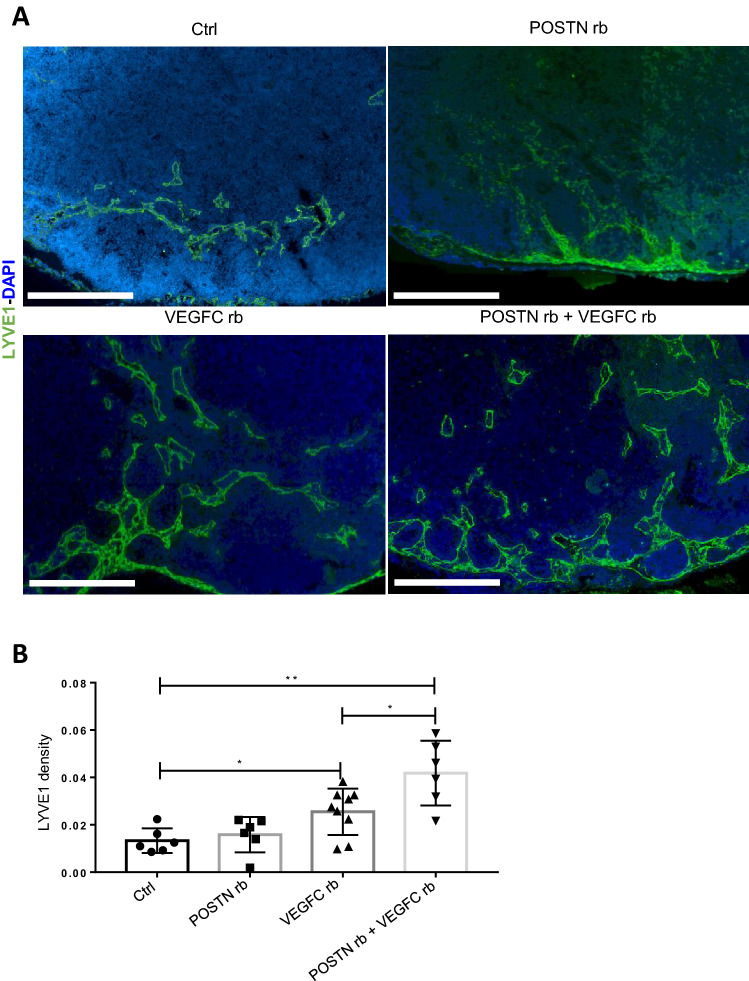
POSTN boosts in vivo the lymphangiogenic response initiated by VEGF-C*. *Inguinal LNs were injected with recombinant POSTN, VEGF-C or a combination of both for 3 days. **a** Morphometric analysis of LYVE1 lymphatic vessels (in green) in inguinal LNs 3 days after injection. Bars = 250 µm. **b** Scatter graph uses scatter plots to represent LYVE1 density assessed by a computer assisted method. Results are expressed as mean ± SD (*n* ≥ 6), and statistical analyses were performed using a Wilcoxon–Mann–Whitney test (*p < 0.05, **p < 0.01)

To further confirm the impact of POSTN on lymphangiogenesis, recombinant POSTN was injected, as described above, with or without VEGF-C in inguinal LNs (Fig. 5a). The injection of recombinant POSTN alone had no impact on lymphatic vessel development (Fig. 5a), whereas recombinant VEGF-C induced an increase in LYVE1 density. Notably, the combination of POSTN and VEGF-C stimulated even more the lymphangiogenic response (Fig. 5b). These data demonstrated that in vivo*,* POSTN alone is not sufficient to per se induce lymphangiogenesis, but actually boosts the lymphangiogenic response initiated by VEGF-C.

### POSTN is increased in locally advanced cervical cancer

**Fig. 6:**
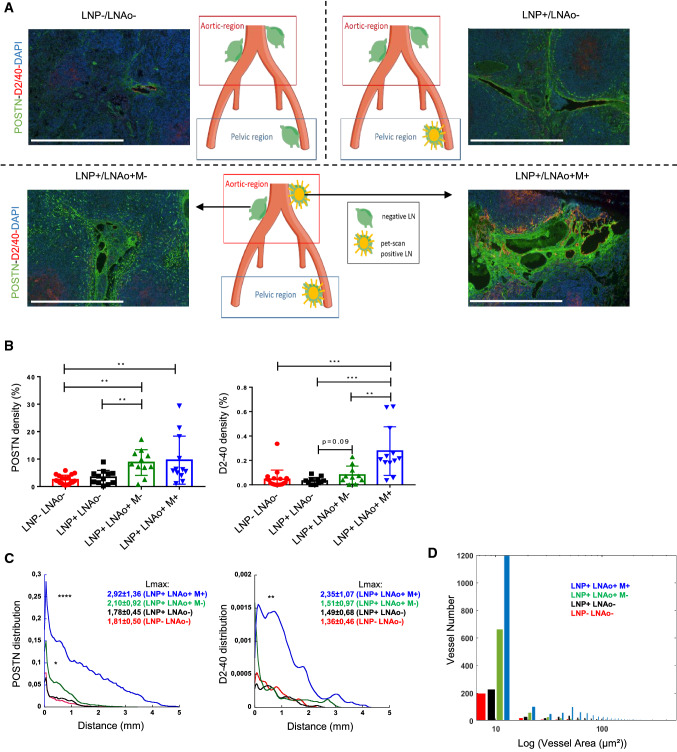
POSTN is increased in locally advanced cervical cancer. Analysis of POSTN and lymphatic network in patients with no LN metastasis (LNP−/LNAo−), one metastatic pelvic LN but none in para-aortic (LNP+/LNAo−) and pelvic and para-aortic metastatic LNs (LNP+/LNAo+ M+). In this group, the surrounding negative para-aortic LN was analyzed (LNP+/LNAo+ M−) as presented in the schema in which, green and yellow LNs correspond to the pet-scan status (negative in green or positive in yellow). **a** Double immunostaining of POSTN (green), Podoplanin (D2-40) (red) and nuclei (DAPI, blue). Scale bars represent 500 µm. **b** A computer-assisted analysis of POSTN and Podoplanin densities (in percentage) in LNP−LNAo− (red), LNP+LNAo− (black), LNP+LNAo+ (green) and LNP+LNAo+M+ (blue). Graphs are presented as mean ± SD and statistical analyses were performed using a Wilcoxon–Mann–Whitney test: (p* < 0.05; p** < 0.01; p*** < 0.001). **c** Spatial lymphatic vessel and POSTN distribution from tissue edge to tissue center measured on whole tissues of LNP−LNAo−, LNP+LNAo−, LNP+LNAo+ and LNP+LNAo+M+. Statistical analyses were performed using a Kolmogorov Smirnov test (*p < 0.05, **p < 0.01, ****p < 0.0001). Maximum distance of migration from the tissue border (Lmax) is indicated and expressed as mean ± SD. (p* < 0.05; p** < 0.01; p**** < 0.0001). **d** Lymphatic vessel size distribution in of LNP-LNAo− (red), LNP+LNAo− (black), LNP+LNAo+ (green) and LNP+LNAo+M+ (blue)

We next extended our analysis to para-aortic LNs (LNAo) of 45 patients with LACC. The patients and their corresponding LNAo were divided into 4 groups according to the pre-treatment PET-Scan signal at the pelvic (LNP) and the para-aortic (LNAo) level (Fig. 6A), as follows:

1. Group 1 (LNP-/LNAo-): LNAo from patients with negative pelvic and para-aortic LNs at PET-Scan.
2. Group 2 (LNP+/LNAo−): LNAo from patients with positive pelvic, but negative para-aortic LNs at PET-Scan.
3. Group 3 (LNP+/LNAo+ M−): LNAo which does not display metastasis from patients with positive PET-Scan signals in LNs of the pelvic and the para-aortic regions.
4. Group 4 (LNP+/LNAo+ M+): a metastatic LNAo from patients with positive PET-Scan signals in LN of the pelvic and the para-aortic regions.

Lymphatic vessel and POSTN densities were low and not statistically different in PET-scan negative LNAos whatever the status of the pelvic LNs (groups 1 and 2) (Fig. 6b). A significant POSTN deposit and increased lymphangiogenesis were found in metastatic (M+) LNAos when both the pelvic nodes and LNAos were positive (group 4) (Fig. 6A). Interestingly, in those patients, the surrounding PET-SCAN negative LNAos (group 3) (LNP+/LNAo+ M−) displayed an increased deposit of POSTN, while the lymphatic vessel density was unchanged (Fig. 6b), suggesting that POSTN production occurs prior to lymphangiogenesis initiation. This observation was further supported by determining the spatial distribution of POSTN and lymphatic vessels in LNAos. Both POSTN and lymphatic vessels were detected deeper in the LN paracortex and medulla of the metastatic LNs (group 4, LNP+/LNAo+ M+) compared to the other LN groups. Indeed, the distance between the LN border and the deeper detection of POSTN or lymphatic vessels (Lmax) was higher in the LN of the metastatic groups (2.92 ± 1.36 mm for POSTN; 2.35 ± 1.07 mm for lymphatics) compared to the negative pelvic group (1.81 ± 0.50 mm for POSTN; 1.36 ± 0.46 mm for lymphatics) (*p < 0.05). The area of the lymphatic vessels was also measured with an increased in LNP+/LNAo+ M+ (Fig. 6d) being observed. Similarly to the global density measurements, in non-metastatic LNAos (group 3, LNP+/LNAo+ M−), a significant enhancement of POSTN deposition was detected, while limited superficiel changes in the distribution of lymphatic vessels was seen (with a peak appearing between 0–1) (Fig. 6c). These data demonstrated the potential of POSTN to specifically detect early LN remodeling.

## Discussion

LN remodeling at a pre-metastatic or a metastatic stage is a dynamic and complex process, which is still mechanistically poorly understood. In this study, using both clinical and experimental LN samples, it was demonstrated that POSTN deposition is associated with a profound remodeling of the lymphatic vasculature. Evidence was provided that POSTN boosts the lymphangiogenic response initiated by VEGF-C and promotes the subsequent LN colonization by metastatic cells. The functional implication of POSTN on these important biological processes taking place during the metastatic cascade was illustrated in two murine models (ear sponge assay and intra-nodal injection of tumor cells) using neutralizing anti-POSTN antibodies and the recombinant protein.

POSTN is an ECM protein, which plays a key role in tissue remodeling by interacting with various ECM proteins such as collagen, fibronectin and integrins [15]. POSTN is highly expressed in primary tumors and has been reported to be upregulated in lung and liver PM niche [21, 22]. That said, its implication in the LN PM niche remains elusive although it has recently been identified in metastatic LNs from patients with squamous cervical cancer [24]. Through proteomic analysis and immunostaining, the first evidence is provided that POSTN is overproduced in the PM SLN of patients with early cervical cancer. Our data support the fact that this matricellular protein plays an important role in the lymphangiogenic process and in the metastatic LN colonization. Accordingly, the administration of anti-POSTN blocking antibody successfully reduced the number and size of LN metastases, which is in line with other previous studies reporting that the downregulation of POSTN in PM lungs led to a reduction in metastases area [38]. A key finding here is the association between POSTN deposition and the lymphangiogenic response. An increased deposit of POSTN and a denser lymphatic network, was observed, initially at the periphery of LN at pre-metastatic stages, and subsequently deeper inside the organ at more advanced or metastatic stages. Notably, the colocalization between POSTN and lymphatic vessels progressively increased during the PM niche formation in the murine model. In vitro, recombinant POSTN promoted LEC adhesion, spreading, migration and proliferation. In vivo*,* the intra-nodal injection of recombinant POSTN alone did not affect the lymphatic vasculature. However, the co-injection of VEGF-C and POSTN led to a higher lymphangiogenic response than an exclusive administration of VEGF-C. These data support the innovative concept that POSTN does not induce lymphangiogenesis per se, but rather promotes the lymphangiogenic response initiated, at least by VEGF-C. Accordingly, in the murine model, the lymphangiogenic response preceded the POSTN deposition. Cancer-associated fibroblasts (CAFs) are recognized as an important source of POSTN in liver and colorectal cancers [39, 40], and in metastatic LNs of patient with cervical cancer [24]. POSTN production might be induced by several tumor-derived factors including TGF-β [41, 42]. In metastatic lungs, CAFs have been reported to produce POSTN in response to TGF-β released by infiltrating tumor cells [43]. In addition, IL6 produced by inflammatory and tumor cells is able to induce POSTN production by CAFs via STAT3 signaling [44]. Following in situ hybridization performed on human LN samples it was also found that αSMA+ fibroblasts produce POSTN. This result was confirmed in vitro by the significant secretion of POSTN in the medium and its deposition into the matrix by FRC in culture, as assessed by ELISA and immunostaining of cell-derived matrix, respectively. In contrast, LECs did not produce this protein or only in insignificant quantities. POSTN production was increased following stimulation by VEGF-C and by medium conditioned by tumor cells. A correlation between POSTN and VEGF-C expression has already been reported in patients with head and neck cancer [45]. Our data demonstrated that tumor-derived factors, among which VEGF-C likely plays a key role, can activate LEC and induce POSTN deposition by FRC, which in turn further promotes lymphangiogenesis. This interplay between POSTN and lymphatic vasculature seems to be unique since POSTN was not involved in the augmentation of HEV structures.

An additional finding of interest relies on the fact that POSTN could be a potential marker of cervical cancer progression by its expression in early stages of the metastatic cascade and its association with lymphatic vessels. The lymphatic dissemination of cancer cells is well documented and an increase of lymphatic vessel density both in the primary tumor and in LN is associated with a high risk of LN dissemination [1, 2, 46]. In clinical practice, LN status is still based on the histological detection of metastatic cells, requiring an in-depth analysis of serial LN sections to avoid missing metastatic cells. The identification of reliable markers is thus mandatory to facilitate and improve LN staging, although the quantification of lymphatic density is relatively difficult for routine analysis. The analysis of a lymphatic network associated with POSTN could be a better way to identify early changes in a LN microenvironment. The study of the spatial distribution highlighted that the main lymphatic and POSTN remodeling occur in the periphery of the LN. Interestingly, a correlation between POSTN and lymphatic vessels was observed in metastatic LN in early stages of the metastatic cascade highlighting the prognostic interest of evaluating POSTN and lymphatic vessels co-staining. Further studies on larger patient cohorts are therefore recommended to establish the prognostic value of such LN remodeling. This concept is in line with the over-expression of POSTN observed in a number of cancers (such as prostate, lung, pancreatic, breast and liver cancer) [47, 48], and the correlation established between POSTN expression in the primary tumor and a poor prognosis [20, 49]. It is worth noting that most of the human studies related to LN PM niches, focus on the first draining LN. Advantage was taken of the features of human cervical cancers that progress from early to advanced cancer stages with a progressive extension of metastases from pelvic to para-aortic LNs. Although it is believed that LN architecture alterations are most likely induced by lymphangiogenic factors drained from the primary tumor, our data did not confirm a systemic dissemination of those factors affecting globally the LNs network. In contrast, a tissue remodeling was only noted in para-aortic LNs when a pelvic LN was PET-Scan positive. This unprecedented observation supports a cascade of LN remodeling from one LN to the surrounding draining ones rather than a concomitant activation of all LNs in the whole basin. This specific process might involve the release of factors or exosomes from the primary tumor to pelvic LNs, and thereafter from metastatic LNs to the next ones, that would then bind to specific cells or structures in the draining LN.

Therapeutic strategies on targeting lymphangiogenesis are not yet conclusive. For example, both pre-clinical and clinical data have failed to demonstrate the efficacy of VEGF receptor inhibitors on LN metastases [50, 51]. The experimental demonstration that LN metastasis can feed the dissemination to distant organs [6, 7] demonstrated that LN PM niche and LN metastasis are worth treating although POSTN has been reported to promote tumor cell proliferation, migration and invasion. Our study supports the fact that functional POSTN implication in lymphangiogenesis and POSTN-blocking antibody drastically reduces LN colonization by metastatic cells. In a recent study [24], POSTN was also shown to disturb the lymphatic barrier via VE-Cadherin pathway, thereby promoting LN colonization [24]. All these data highlight the therapeutic potential of inhibiting POSTN in LN, with or without being combined with anti-lymphangiogenic agents, which would provide effective therapies. Thus, targeting POSTN is worth considering to prevent not only lung [43], but also LN metastasis.

POSTN has also been discovered in cancer-derived exosomes [52–54] and was enriched in exosomes secreted from metastatic tumor cells and in plasma samples from patients with LN metastasis [53]. Recently, a comparison between lymphatic exudate contents from metastatic and non-metastatic melanoma patients showed an increase in the number of exosomes and a different protein expression profile [55]. These data suggest that exosomes from metastatic and non-metastatic melanoma express protein signatures that correlate with different stages of the metastatic process. The potential for certain tumors to metastasize to specific organs, was initially put forward by Stephan Paget but the mechanisms underlying this process remain unknown. Tumor-derived exosomes expressing particular integrin and ECM molecules, partially dictated future PM sites such as lung and liver [52]. Studies on the role of exosomes in promoting metastasis have also revealed that lung, liver, bone, and LN PM niche display unique features [52]. Unfortunately, the PM niche concept is not yet used in a clinic setting and only the presence or absence of metastatic cells in LN is verified as a prognostic factor. The use of new markers such as POSTN could pave the way to new approaches in blocking cancer progression. Collectively, our data provide new insights into the LN PM niches with POSTN as a driver of LN metastasis.

### Supplementary Information

Below is the link to the electronic supplementary material.

Supplementary file1 (PDF 2324 KB)

## Data Availability

The datasets generated during and/or analysed during the current study are available from the corresponding author on reasonable request.
