# Supplementary material for: Periostin in lymph node pre-metastatic niches governs lymphatic endothelial cell functions and metastatic colonization
Source: Cell Mol Life Sci. 2022 May 14;79(6):295. doi: 10.1007/s00018-022-04262-w (PMC9107454; doi:10.1007/s00018-022-04262-w)
Supplement: Supplementary file 1 — Supplementary file1 (PDF 2324 KB) [file 18_2022_4262_MOESM1_ESM.pdf]

Periostin in lymph node pre-metastatic niches governs lymphatic endothelial cell functions and metastatic colonization

Gillot Lionel[1], Lebeau Alizée[1], Baudin Louis[1], Pottier Charles[1], Louis Thomas[1], Durré Tania[1], Longuespée Rémi[2], Mazzucchelli Gabriel[3], Nizet Christophe[4], Blacher Silvia[1], Kridelka Frédéric[1,5,#], Noël Agnès[1,6,#].

[1] Laboratory of Tumor and Development Biology, GIGA-Cancer, Liege University, B23, Avenue Hippocrate 13, Sart-Tilman, B-4000, Liege, Belgium

[2] Department of Clinical Pharmacology and Pharmacoepidemiology, Heidelberg University Hospital, Im Neuenheimer Feld 410, 69120 Heidelberg, Germany

[3] GIGA Proteomics Facility, Liège University, Sart-Tilman, B-4000, Liège, Belgium

[4] Department of Plastic and Reconstructive surgery, CHU Liege, Sart-Tilman, 4000, Liege, Belgium

[5] Department of Obstetrics and Gynecology, CHU Liege, Sart-Tilman, 4000, Liege, Belgium

[6] Walloon Excellence in Lifesciences and Biotechnology (WELBIO), Liege University, Belgium

# co-senior authors

Corresponding authors: Agnès Noël**,** Laboratory of Tumor and Development Biology, GIGA-Cancer, Liege University, B23, Avenue Hippocrate 13, Sart-Tilman, B-4000, Liege, Belgium (Phone number: +32 4 366 25 69)
Email Address**:** agnes.noel@uliege.be

Supplementary figures:

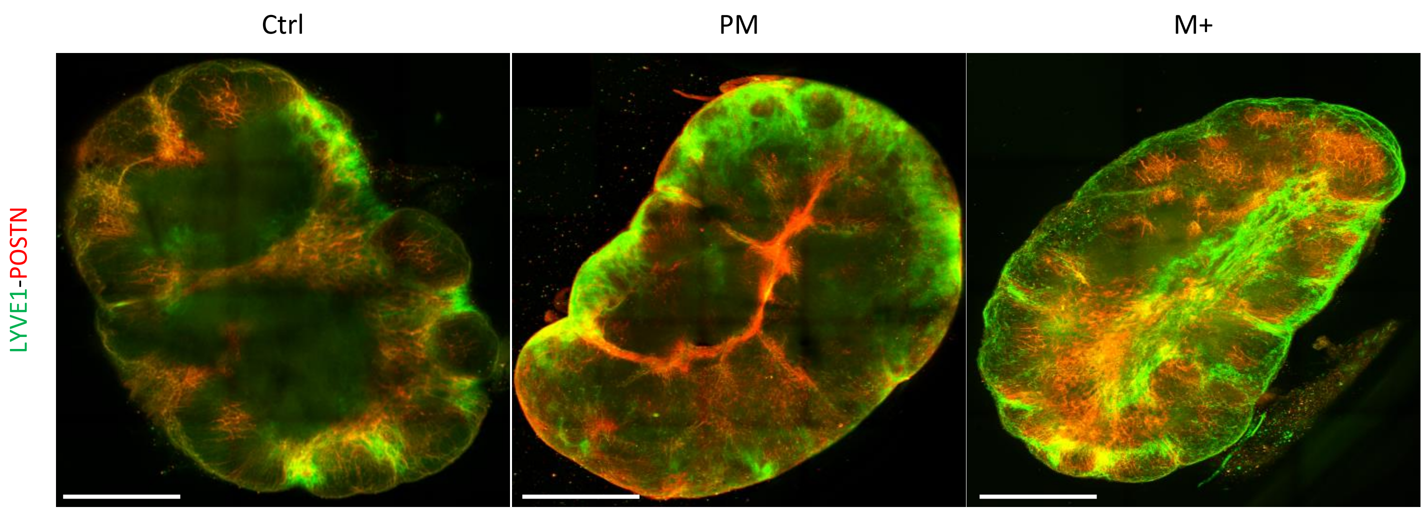

Supplementary Figure 1: 3D analyzes of the POSTN network and lymphatic vessels. Immunofluorescence of the complete LN at pre-metastatic (PM) (2 weeks) and metastatic (M+) (4 weeks post implantation) stages compared to ctrl (POSTN in red and LYVE1 in green). Bars = 500 μm.

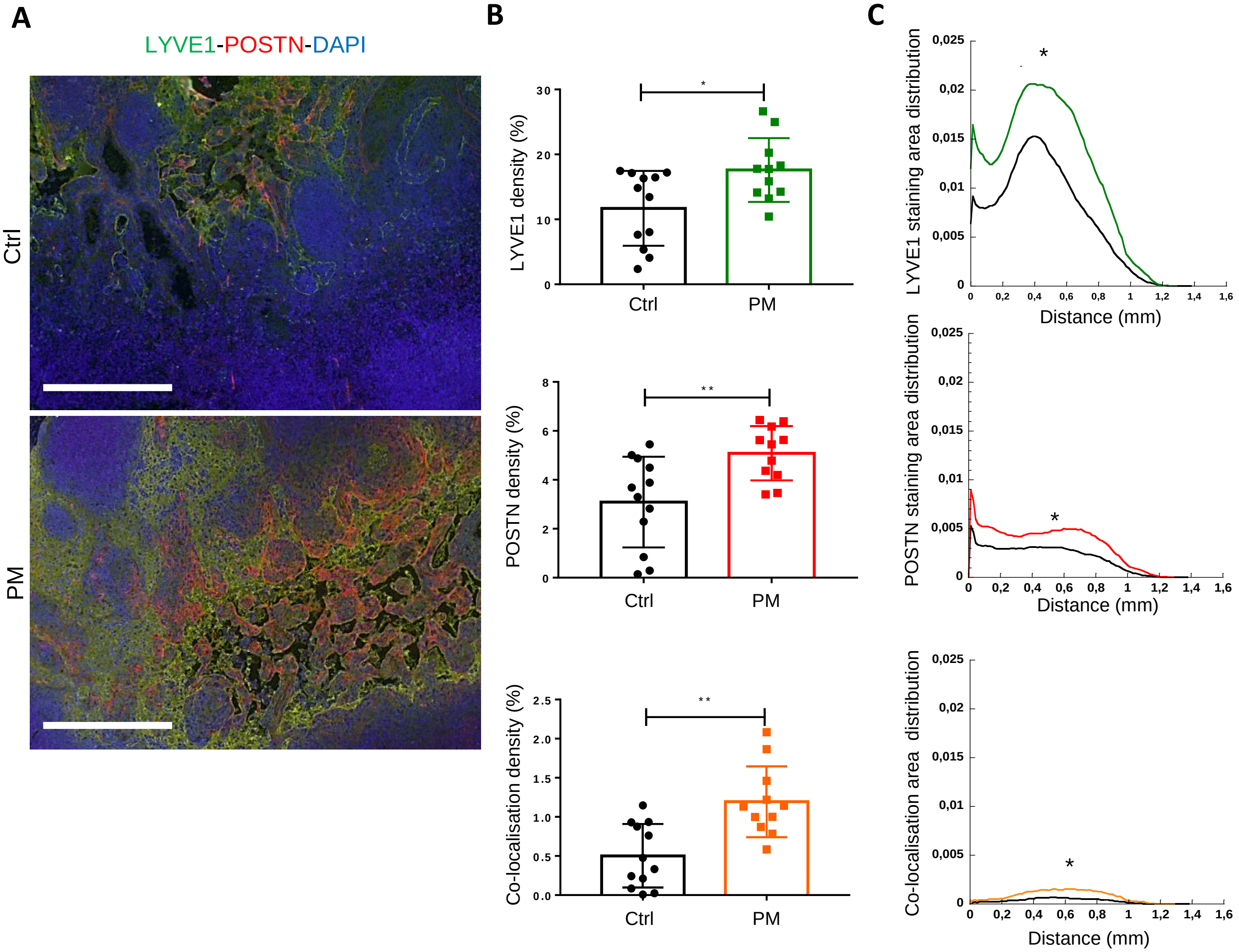

Supplementary Figure 2: POSTN is increased in pre-metastatic LNs of mice implanted with CaSki cells. Morphometric analysis of POSTN and LYVE1 lymphatic vessels in pre-metastatic LN as compared to the control. **A)** POSTN, LYVE1 and cell nuclei were stained in red, green and blue respectively in the control and pre-metastatic (PM) stage. Bars = 250 µm. **B)** Scatter graphs use scatter plots to represent POSTN, LYVE1 and colocalization density (in percentage) assessed by a computer assisted method ($n \geq 9$). Results are expressed as mean ± SD, and statistical analyses were performed using a Wilcoxon-Mann-Whitney test (*p<0,05; **p<0,01). **C)** Spatial distribution analysis of LYVE1, POSTN, and the colocalization of both stainings from tissue edge to tissue center. Statistical analyses were performed using a Kolmogorov Smirnov test (*p<0.05). **D)** qRT-PCR performed on E6-E7 HPV proteins from LNs samples. The primary tumor was used as a positive control.

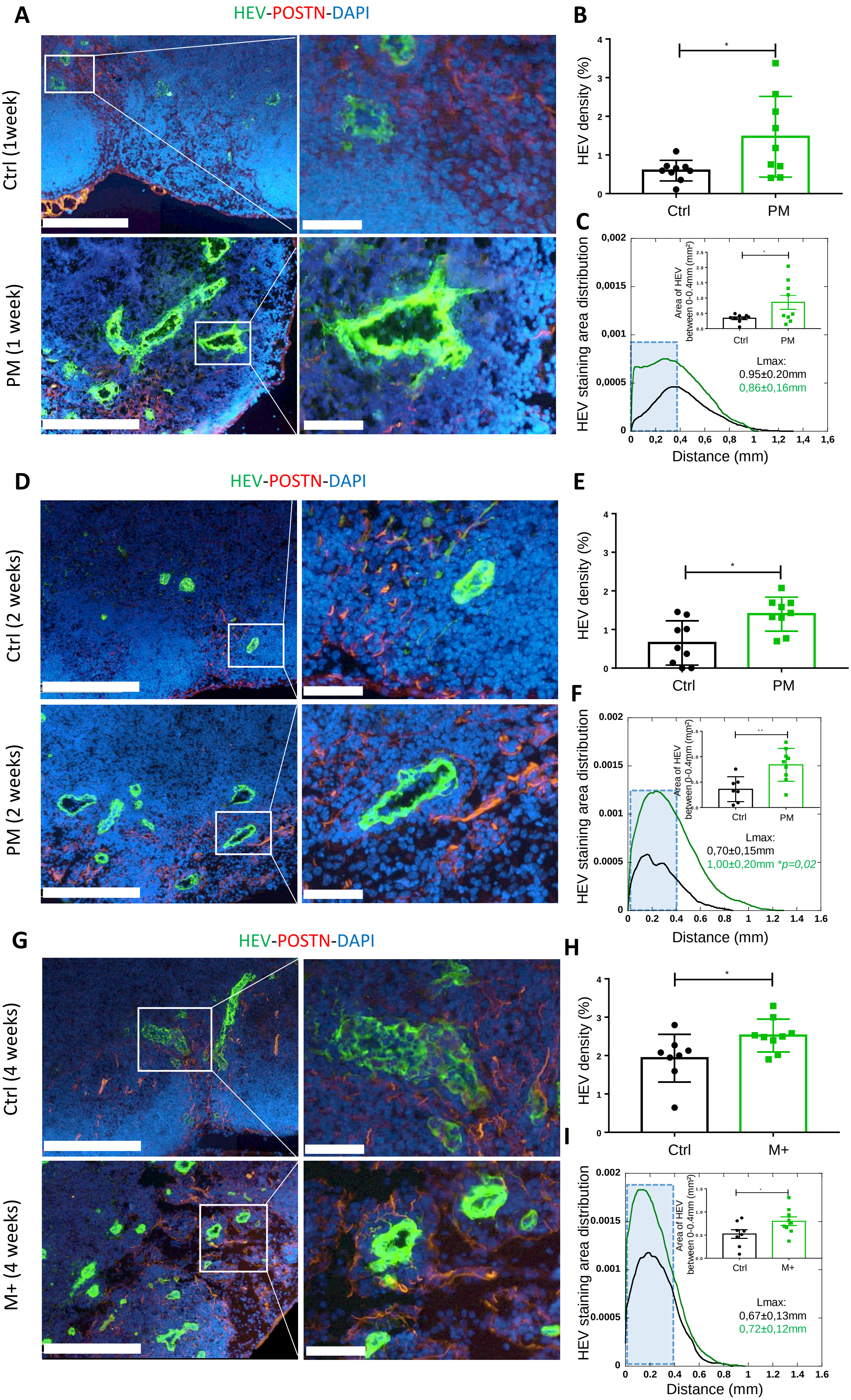
A
HEV-POSTN-DAPI
Ctrl (1week)
PM (1 week)
B
HEV density (%)
Ctrl
PM
C
HEV staining area distribution
Area of HEV between 0-0.4mm (mm²)
Lmax:
0.95±0.20mm
0,86±0,16mm
Distance (mm)
D
HEV-POSTN-DAPI
Ctrl (2 weeks)
PM (2 weeks)
E
HEV density (%)
Ctrl
PM
F
HEV staining area distribution
Area of HEV between 0-0.4mm (mm²)
Lmax:
0,70±0,15mm
1,00±0,20mm *p=0,02
Distance (mm)
G
HEV-POSTN-DAPI
Ctrl (4 weeks)
M+ (4 weeks)
H
HEV density (%)
Ctrl
M+
I
HEV staining area distribution
Area of HEV between 0-0.4mm (mm²)
Lmax:
0,67±0,13mm
0,72±0,12mm
Distance (mm)

Supplementary Figure 3: POSTN is not associated with HEVs in (pre)-metastatic LNs. Morphometric analysis of experimental (pre)-metastatic LNs as described in Figure 2. **A-D-G)** POSTN (red) and HEV (MECA79+ in green) stainings in pre-metastatic (PM) (at 1 week in A and 2 weeks in D) and in metastatic (M+) (at 4 weeks, G). Bars = 250 µm and 50 µm in the right (higher magnification of the insert images). **B-E-H)** Scatter graphs use scatter plots to represent POSTN and HEV densities (in percentage) (n ≥ 9). Results are expressed as mean ± SD (Wilcoxon-Mann-Whitney test: *p<0,05). **C-F-I)** Spatial distribution analysis from tissue edge to tissue center. The blue rectangle delineates the area between 0-0.30 mm from the LN border where the cumulate normalized areas of HEV and POSTN were measured and represented in the top right. Maximum distance of migration from the tissue border (Lmax) is indicated. Results are expressed as mean ± SD (Wilcoxon-Mann-Whitney test: *p<0,05; **p<0,01). **D-I)** All the results are representative of two independent experiments.

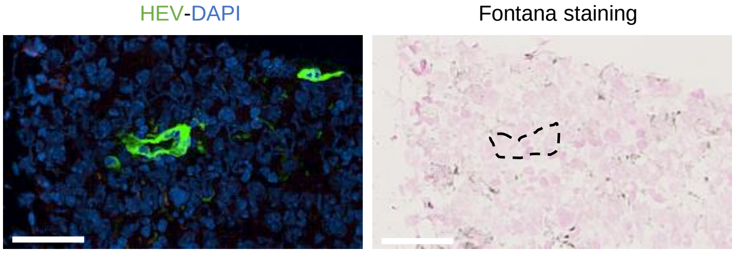

Supplementary Figure 4: HEVs are observed inside the tumor area. HEV and cell nuclei were stained in green and blue, respectively. Fontana staining was used to stain tumor cells in metastatic (M+). Bars = 50µm.

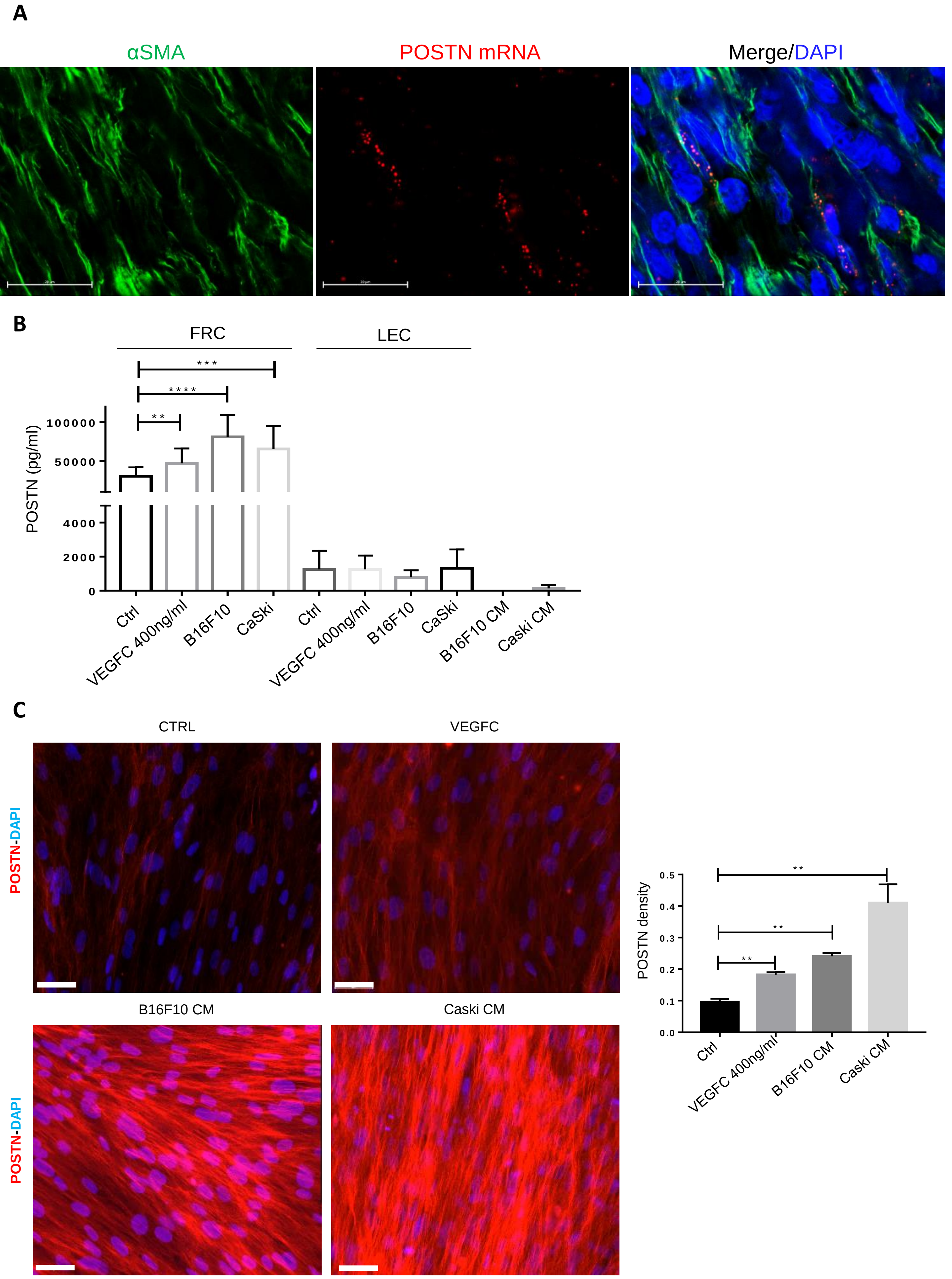
A
αSMA
POSTN mRNA
Merge/DAPI
B
FRC
LEC
POSTN (pg/ml)
100000
50000
4000
2000
0
***
****
**
Ctrl
VEGFC 400ng/ml
B16F10
CaSki
Ctrl
VEGFC 400ng/ml
B16F10
CaSki
B16F10 CM
Caski CM
C
CTRL
VEGFC
B16F10 CM
Caski CM
POSTN-DAPI
POSTN-DAPI
POSTN density
0.5
0.4
0.3
0.2
0.1
0.0
**
**
**
Ctrl
VEGFC 400ng/ml
B16F10 CM
Caski CM

Supplementary Figure 5: POSTN is produced by fibroblasts. A) Representative POSTN (red) mRNA detection by RNAscope and immunostaining of α-SMA (green) on human LN sections (scale bar=20 µm). Cell nuclei are stained with DAPI in blue. **B)** ELISA of human POSTN performed on FRCs and LECs stimulated by VEGF-C (400ng/ml) or medium conditioned (CM) by B16F10 or CaSki cells (with at least 3 wells per condition in each experiment). Results (pg/ml) are expressed as mean ± SD (Mann-Whitney test: $^{**}p<0.01$; $^{***}p<0.001$ $^{****}p<0.0001$). Results represent a set of three independent experiments. **C)** POSTN immunostaining of FRC (in red) stimulated by VEGF-C (400ng/ml), B16F10 CM or CaSki CM. Nuclei were counterstained with DAPI (blue). Bars=50 µm. Results are expressed as mean ± SD and represent a set of two independent experiments (Mann-Whitney test: $^{**}p<0.01$).

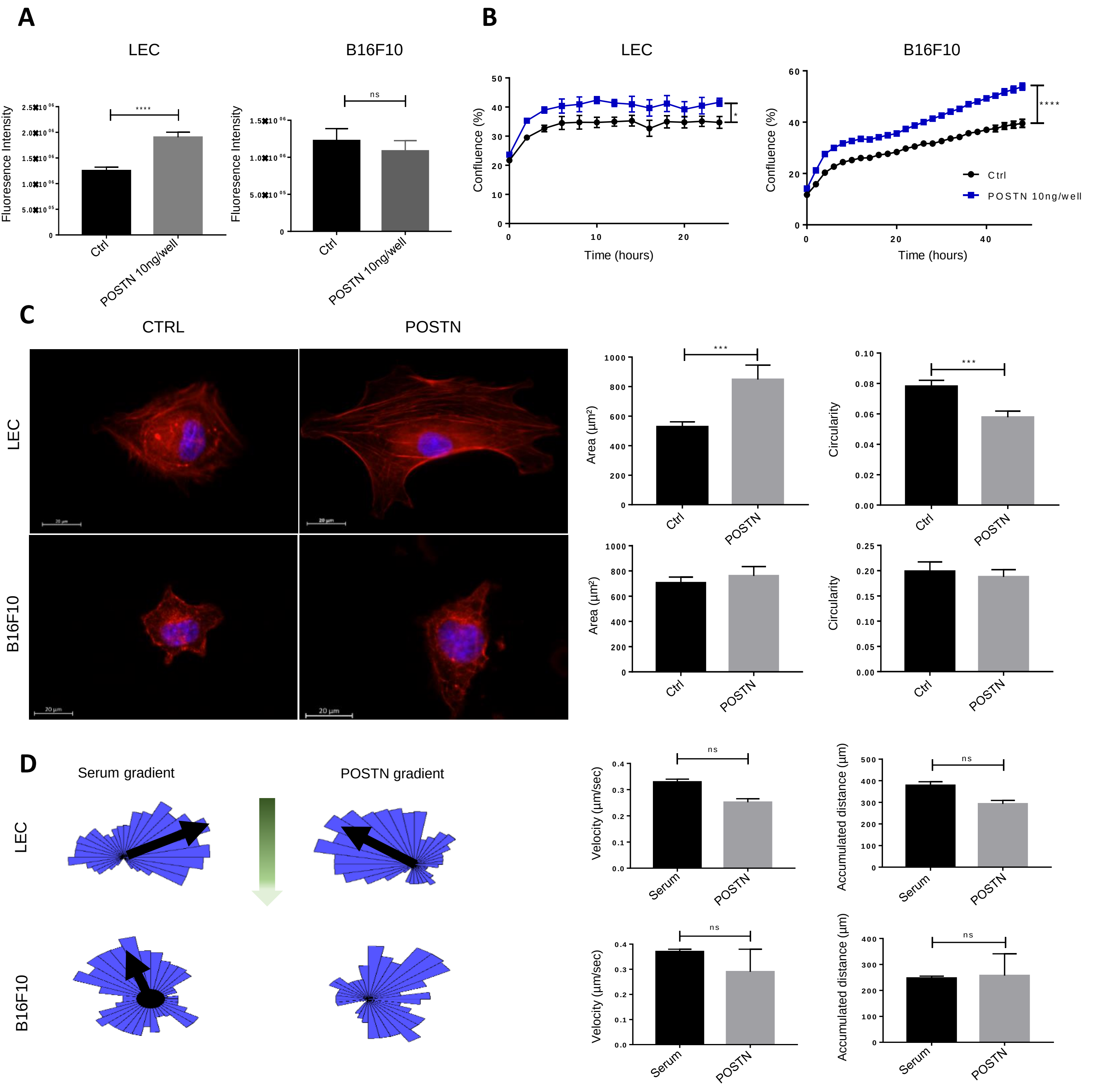
A
LEC
B16F10
Fluoresence Intensity
Fluoresence Intensity
****
ns
Ctrl
POSTN 10ng/well
B
LEC
B16F10
Confluence (%)
Time (hours)
*
****
Ctrl
POSTN 10ng/well
C
CTRL
POSTN
LEC
B16F10
Area (µm²)
Circularity
***
Ctrl
POSTN
D
Serum gradient
POSTN gradient
LEC
B16F10
Velocity (µm/sec)
Accumulated distance (µm)
ns
Serum
POSTN

Supplementary Figure 6: POSTN exerts different effects on LECs and tumor cells

A) Adhesion assay of LECs (left panel) and B16F10 cells (right panel) on POSTN coated-wells. Results represent a set of three independent experiments (with 12 wells per condition in each experiment) and are expressed as mean ± SD (Mann–Whitney test: ****$p<0.0001$). **B)** LEC and B16F10 proliferation analysis using the IncuCyte S3 system. LECs and B16F10 cells were seeded on a POSTN-coated well for 24h and 48h, respectively. The quantification is represented by the percentage of confluence. Results are expressed as mean ± SEM and the Anova two-way significance test was used (* $p < 0.05$, ** $p < 0.01$). **C)** Characterization of LEC and B16F10 cell morphology via cell circularity and area quantification in a POSTN-coated well (10ng/well) compared to the control. Cells were stained with phalloidin (red) and DAPI (blue) used as a marker for the nuclei. One representative experiment out of the 3 analyzed more than 20 cells per condition. Bars=20 µm. Statistical analyses were performed using a non-parametric Mann–Whitney test (***$p<0.001$). **D)** Chemotactic response of LECs and B16F10 cells to a gradient of complete medium or POSTN. The rose diagram illustrates the migration direction migration of more than 20 cells. Black arrows indicate the mean migration direction. Histograms represent the migratory speed (µm/min) and the accumulated distance (µm) where results are expressed as mean (n = 3 biological replicates) ± SEM. Statistical analyses were performed using a non-parametric Mann–Whitney test.
